# Study on Stress Development in the Phase Transition Layer of Thermal Barrier Coatings

**DOI:** 10.3390/ma9090773

**Published:** 2016-09-13

**Authors:** Yijun Chai, Chen Lin, Xian Wang, Yueming Li

**Affiliations:** 1 State Key Laboratory for Strength and Vibration of Mechanical Structures, Shaanxi Key Laboratory of Environment and Controlfor Flight Vehicle, Xi’an Jiaotong University, Xi’an 710049, China; cyj1991@stu.xjtu.edu.cn (Y.C.); wangxian@mail.xjtu.edu.cn (X.W.); 2 State Key Laboratory for Manufacturing System Engineering, Xi’an Jiaotong University, Xi’an 710049, China; linchen19870117@stu.xjtu.edu.cn

**Keywords:** thermal barrier coatings (TBCs), constitutive equation, phase transition layer, stress development

## Abstract

Stress development is one of the significant factors leading to the failure of thermal barrier coating (TBC) systems. In this work, stress development in the two phase mixed zone named phase transition layer (PTL), which grows between the thermally grown oxide (TGO) and the bond coat (BC), is investigated by using two different homogenization models. A constitutive equation of the PTL based on the Reuss model is proposed to study the stresses in the PTL. The stresses computed with the proposed constitutive equation are compared with those obtained with Voigt model-based equation in detail. The stresses based on the Voigt model are slightly higher than those based on the Reuss model. Finally, a further study is carried out to explore the influence of phase transition proportions on the stress difference caused by homogenization models. Results show that the stress difference becomes more evident with the increase of the PTL thickness ratio in the TGO.

## 1. Introduction

A thermal barrier coating system (TBCs) is highly advanced material system, mainly applied to the metallic surfaces of gas-turbine engines. These coatings provide heat insulation to the metallic parts from the extremely hot gas steam [1,2,3,4], and allow the engines to work at higher operating temperatures, which could significantly improve the turbine efficiency [5,6]. TBCs mainly consists of the 7 wt % Y_2_O_3_-stabilized ZrO_2_ (7YSZ) ceramic layer, i.e., Top Coat (TC), and the (Ni,Pt)Al or MCrAlY layer, i.e., bond coat (BC). TC is the primary heat-resistant layer with a lower heat conduction coefficient, while the BC is composed of Al and serves to prevent the substrate from oxidation and corrosion, and to improve cohesiveness between the TC and the substrate [7,8].

Due to the large and prolonged heat loads, much attention should be paid to the high-temperature mechanical performance of the TBC materials system, especially the high-temperature oxidation behavior, which could reduce the TBC life drastically and lead to thermal fatigue. As the oxygen diffuses through the TC, the BC materials are gradually oxidized to thermally grown oxide (TGO). Thus, there is a layer appeared along the interface where both BC and TGO materials co-exist, which is defined as the phase transition layer (PTL) [9]. As the BC oxidation develops, the phase transition layer grows down towards the substrate, while the early part of the layer finally becomes a TGO layer. In this study, the BC layer is assumed to be mainly consisted of Al, and the phase transition layer are co-existent Al and Al_2_O_3_. The TGO layer is considered to contain the Al_2_O_3_ layer and PTL.

The growth of the phase transition layer could affect the stress development on the interface, which has a significant effect on the interface degradation at high temperatures [10]. The establishment of constitutive relationships is essential for studying the stress development, so the influence of different constitutive relationships on the stress development is of great interest. Some research has been conducted on the constitutive relationship of the phase transition layer. An oxidation-constitutive framework coupling local expansion and local stresses induced by oxidation was proposed by Busso et al. to describe the effect of the phase transition caused by the gradual oxidation on the constitutive behavior of the phase transition zone [10,11]. Hille [9] described the growth of the TGO–BC mixture zone using an oxygen diffusion–reaction model, and investigated the stress-strain behavior within the mixture zone through a constitutive equation based on the Voigt model. It is well known that the composition and microstructure of the materials in TBCs are extremely complicated because of the intricate processes such as local oxidation, diffusion, creep, and sintering of the ceramic material [11]. Therefore, it is difficult to precisely describe constitutive behavior within the PTL and to evaluate the stress by using a specific constitutive formulation. Thus, it would be much more useful if the stress limits based on different constitutive models can be predicted. The Voigt and Reuss model are confirmed as the upper and lower bound among homogenization models [12]. Therefore, the above two models are applied in this study to explore the interface stress.

This work aims at investigating the influence of different homogenization models on stress prediction in TBCs. Thus, a constitutive equation based on the Reuss model is proposed. By using the constitutive equation based on the Voigt model [9] as well, this work studies the stress development in the PTL in detail. Both of the equations are discretized with the finite element method (FEM) and are implemented with the help of the commercial FE package ABAQUS. In the numerical examples, the effect of oxygen diffusion on the growth of the phase transition layer is first explored. The stress development in PTL is then predicted with the two constitutive equations, respectively, and the influence of phase transition proportions on the stresses is investigated. The results may show a reference for further research on the stress development in thermal barrier coatings.

## 2. Theoretical Models

### 2.1. Diffusion-Oxidation Reaction Model for the TGO Layer

Fick’s law is employed in this work, which allows the description of the phenomenon of gas diffusion in a macroscopic point of view:

(1) $${\dot{c}}_{o^{2-}}=Ddiv(\nabla c_{o^{2-}})$$

where $c_{o^{2-}}$ is the concentration of oxygen and *D* is the diffusion coefficient of oxygen. During the oxidation of TBCs, the formation of TGO continuously consumes oxygen. To take this consumption effect into account, a diffusion-oxidation reaction model [13] can be written as follows by using the concept of alkali-silica reaction:

(2) $${\dot{c}}_{O^{2-}}=Ddiv(\nabla c_{O^{2-}})-S(c_{o^{2-}})$$

where $S(c_{o^{2-}})=-A\dot{\xi }$ is the oxidation term and *A* denotes the amount of oxygen consumed by per unit reference volume metal during oxidation. $\dot{\xi }$ is the changing rate of the Al_2_O_3_ dimensionless molar fraction ξ, which can be expressed as:

(3) $$\dot{\xi }=\gamma (1-\xi )c_{o^{2-}}$$

where γ is a constant.

With the diffusion-oxidation reaction model mentioned above, the growth of the phase transition layer can be predicted.

### 2.2. The Constitutive Equation for the Phase Transition Layer

As two limiting cases in the homogenization schemes, the Voigt and Reuss models represent the upper and lower bounds, respectively [12]. In this section, a new constitutive equation based on the Reuss model is proposed to describe the material behavior of the PTL.

#### 2.2.1. Constitutive Equation based on Reuss Model

According to the Reuss model [14], the iso-stress assumption is set as follows:

(4) $$\sigma =\sigma_{\alpha }=\sigma_{\beta }$$

(5) $$\varepsilon =\phi \varepsilon_{\alpha }+(1-\phi )\varepsilon_{\beta }$$

In this work, the PTL is composed of Al and Al_2_O_3_, so the *α* and *β* phases are specified as Al_2_O_3_ and Al phases, respectively.

$\varepsilon_{Al}$ is the total strain in the phase of Al, which is divided into the elastic strain $\varepsilon_{Al}^{e}$ and thermal strain $\varepsilon_{Al}^{th}$:

(6) $$\varepsilon_{Al}=\varepsilon_{Al}^{e}+\varepsilon_{Al}^{th}$$

$\varepsilon_{Al_{2}O_{3}}$ is the total strain in the phase of Al_2_O_3_, which is partitioned into the elastic strain $\varepsilon_{Al_{2}O_{3}}^{e}$, the thermal strain $\varepsilon_{Al_{2}O_{3}}^{th}$ and the eigenstrain $\varepsilon_{Al_{2}O_{3}}^{g}$ due to phase transition obtained from the Pilling-Bedworth ratio [15]:

(7) $$\varepsilon_{Al_{2}O_{3}}=\varepsilon_{Al_{2}O_{3}}^{e}+\varepsilon_{Al_{2}O_{3}}^{th}+\varepsilon_{Al_{2}O_{3}}^{g}$$

Combining Equations (6) and (7), and taking Hooke’s law into account for each phase:

(8) $$\varepsilon_{Al}^{e}=S_{Al}\sigma_{Al}$$

(9) $$\varepsilon_{Al_{2}O_{3}}^{e}=S_{Al_{2}O_{3}}\sigma_{Al_{2}O_{3}}$$

where ***S*** is the flexibility matrixes.

The relationship of stress and strain in the phase transition layer can be written as:

(10) $$\left[(1-\xi )S_{Al}+\xi S_{Al_{2}O_{3}}]\sigma =[\varepsilon -(1-\xi )\varepsilon_{Al}^{th}-\xi (\varepsilon_{Al_{2}O_{3}}^{th}+\varepsilon_{Al_{2}O_{3}}^{g})\right]$$

where ξ is the dimensionless molar fraction of Al_2_O_3_ phase, which values from 0 to 1.

In Equation (10), an equivalent flexibility matrix $S_{eff}$ is defined, which can be expressed as:

(11) $$S_{eff}=(1-\xi )S_{Al}+\xi S_{Al_{2}O_{3}}$$

The constitutive relationship based on the Reuss model can be finally obtained as follows:

(12) $$\sigma ={S_{eff}}^{-1}:[\varepsilon -(1-\xi )\varepsilon_{Al}^{th}-\xi (\varepsilon_{Al_{2}O_{3}}^{th}+\varepsilon_{Al_{2}O_{3}}^{g})]$$

#### 2.2.2. Constitutive Equation based on the Voigt Model

Another homogenization method used in this work is the Voigt model. According to [9], the constitutive equation based on the Voigt model is shown as Equation (13):

(13) $$\sigma =(1-\xi )D_{Al}:(\varepsilon -\varepsilon_{Al}^{th})+\xi D_{Al_{2}O_{3}}:(\varepsilon -\varepsilon_{Al_{2}O_{3}}^{th}-\varepsilon_{Al_{2}O_{3}}^{g})$$

## 3. Numerical Examples

This section contains the numerical calculation conditions applied in the finite element analysis, including detailed information about the finite element model of TBCs adopted in the calculations and material parameters for the diffusion-oxidation reaction model and constitutive equations.

### 3.1. Finite Element Model

**Figure 1:**
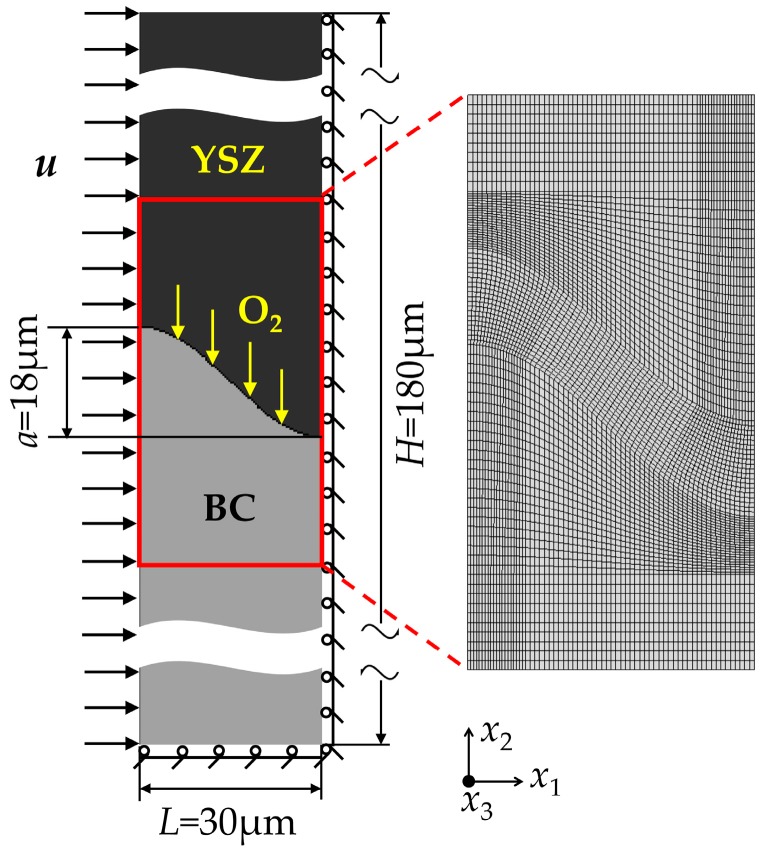
Finite element model of TBCs.

It is assumed that the morphology of the TC-BC interface is idealized as a periodic array of sine interfaces in this paper, as illustrated in Figure 1. The finite element model consists of 14,144 quadratic first-order iso-parametric elements and 14,421 nodes.

The thermal field imposed on TBCs during the service process is assumed to be spatially homogeneous. The TBCs are exposed at the temperature of 1100 °C for 200 h in the service process, and then cools to room temperature (20 °C) during the following 120 s. The TBCs are considered to be stress-free in the initial stage of the oxidation process, and these temperature-converting processes are defined as an operating cycle and applied to the finite element analysis for the evolution of stress.

For the diffusion analysis of this study, the oxygen concentration is considered to be uniform on the interface between TC and BC, and has a constant value of 1.5 mol/m^3^. With the diffusion-oxidation reaction model introduced in Equation (2), oxide growth occurred during the isothermal exposure periods at 1100 °C.

The displacement boundary condition is illustrated in Figure 1. The bottom boundary is constrained in *x*_2_ direction, depending on the fact that the thickness of the substrate is much larger than the TBCs; thus, it can be regarded that there is almost no translation in *x*_2_ direction on the substrate when TBCs deforms during the service process. The right boundary is clamped in the *x*_1_ direction due to the symmetry of the TBCs model. Meanwhile, a uniform displacement *u* is applied at the left boundary in *x*_2_ direction, considering the effect of the thermal contraction imposed by the substrate on TBCs during the cooling stage, which can be obtained by Equation (14):

(14) $$u=\alpha_{sub}(T-T_{ref})L$$

where $\alpha_{sub}$ is the thermal expansion coefficient of the substrate and $T_{ref}$ is the reference temperature of 1100 °C. *L* is the length of the finite element model values 30 μm shown in Figure 1.

In the direction *x*_3_ perpendicular to the *x*_1_-*x*_2_ plane, the deformation of the substrate is imposed on the TBCs by a generalized plane-strain condition:

(15) $$\varepsilon_{33}=\alpha_{sub}(T-T_{ref})$$

### 3.2. Physical and Mechanical Properties

In this study, the TC, the TGO, and the BC are considered as isotropic materials and the Young’s modulus *E*, Poisson’s ratio *ν*, and the thermal expansion coefficient *α* of TC, BC, and TGO phases are listed in Table 1.

**Figure 2:**
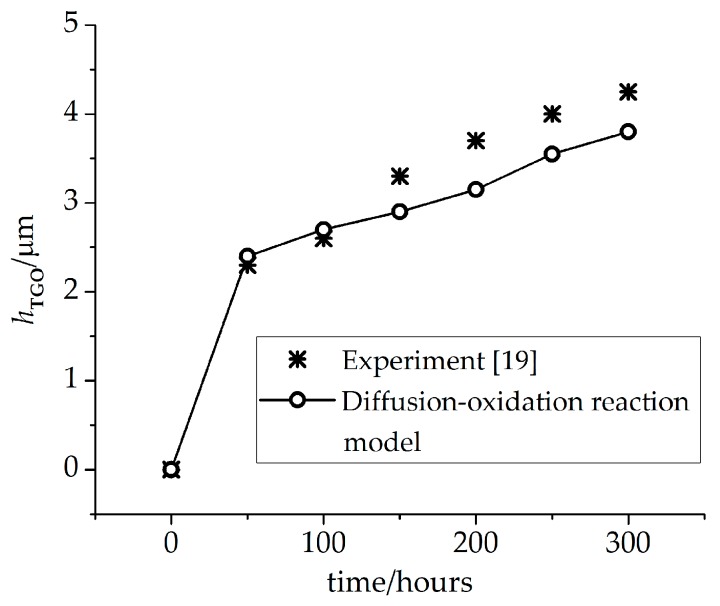
The TGO thickness based on the diffusion-oxidation reaction model after oxidation stage.

The diffusion-oxidation reaction model in Equation (2) is used to predict the oxide growth history. The values of oxygen diffusion coefficients of TGO and BC phase are regarded to be the same [17]. The parameter *A* obtained in [18] is used. The parameter *γ* in Equation (2) can be numerically calculated via the process of TGO formation in the experiment mentioned [19]. These parameters are listed in Table 2. Figure 2 shows the TGO thickness calculated by the diffusion-oxidation reaction model after several hours of oxidation. It can be seen that the numerical results are in good accordance with the experimental results from [19].

## 4. Results and Discussion

**Figure 3:**
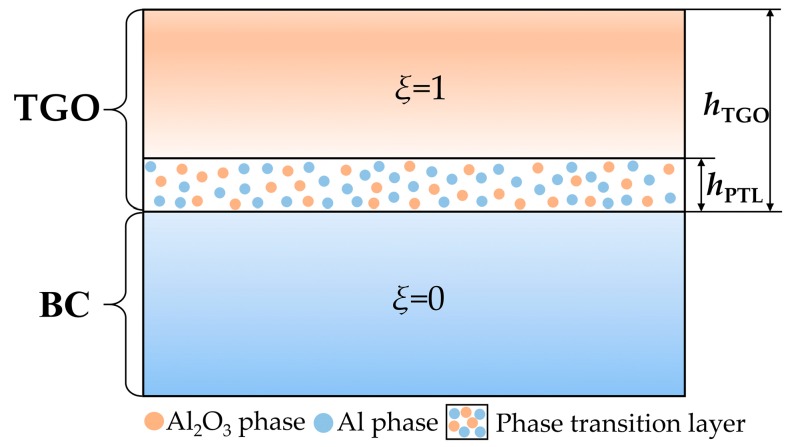
Sketch of the phase transition layer (PTL).

As mentioned in the introduction, an Al and Al_2_O_3_ co-existent region forms over the BC interface during the oxidation procedure, which is defined as the phase transition layer (PTL) with the dimensionless molar fraction of Al_2_O_3_, *ξ*. The purely Al_2_O_3_ layer (*ξ* = 1) and the PTL (0 < *ξ* < 1) compose the TGO layer, as can be seen in Figure 3. In this subsection, the growth of PTL and the stresses in PTL are investigated. In order to explore the effect of PTL growth on the stress development, the term *phase transition proportion* is defined, indicating the thickness ratio of the PTL taking part in the total TGO. The phase transition proportion in this work is expressed as *h*_PTL_/*h*_TGO_ (see Figure 3), where *h*_TGO_ is the total thickness of TGO and *h*_PTL_ is the thickness of the PTL. Each value of the phase transition proportion represents a TGO growth state.

In the simulation, the TBCs exposed to the thermal environment with an oxidation stage at 1100 °C for 200 h, and a cooling stage at 20 °C for 120 s, is analyzed. The diffusion-oxidation reaction model and the constitutive equations introduced above are applied to the numerical calculations.

### 4.1. Growth of the Phase Transition Layer

**Figure 4:**
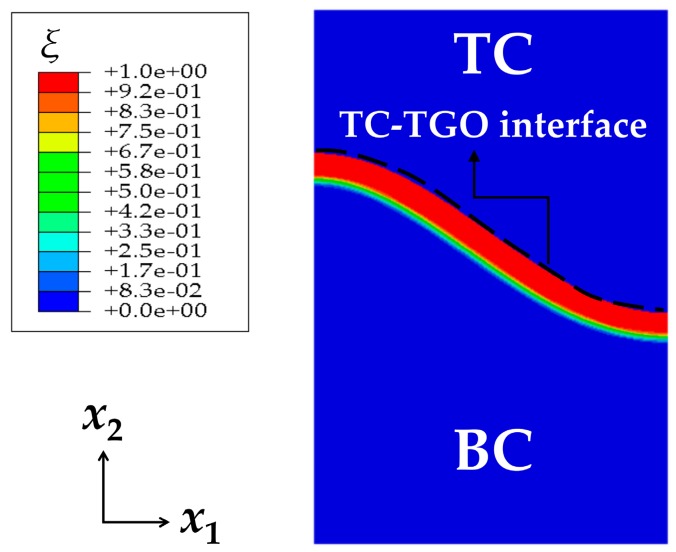
The contour plot of TGO growth after 200 h oxidation (*D* = 3.5 × 10^−14^ m^2^/s).

**Figure 5:**
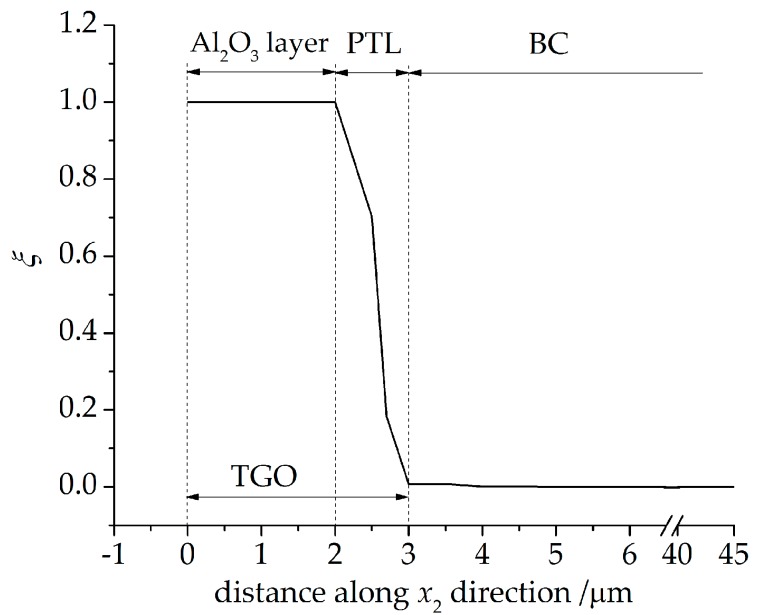
The dimensionless molar fraction of Al_2_O_3_ along *x*_2_ direction at *x*_1_ = 0 after 200 h oxidation (*D* = 3.5 × 10^−14^ m^2^/s).

As discussed earlier, with oxygen diffusing through the TC, the BC is assumed to be oxidized gradually, leading to the formation of the PTL. Figure 4 presents the contour plot of TGO growth after 200 h oxidation with the oxygen diffusion coefficient of 3.5 × 10^−14^ m^2^/s. It shows that Al_2_O_3_ forms on TC-BC interface at the beginning of oxidation. As the oxidation proceeds, Al_2_O_3_ grows uniformly in the direction of BC, and along *x*_2_ axis, the dimensionless molar fraction of Al_2_O_3_ *ξ* decreases. To observe the growth of TGO clearly, *ξ* curve is plotted reflecting *ξ* variation along the *x*_2_ direction at *x*_1_ = 0, as shown in Figure 5. The horizontal axis origin is set to be the point where the TGO emerges firstly in the direction *x*_2_. It can be seen that the thickness of TGO is 3 mm and PTL of 1 mm, with a phase transition proportion *h*_PTL_/*h*_TGO_ of 33%.

**Figure 6:**
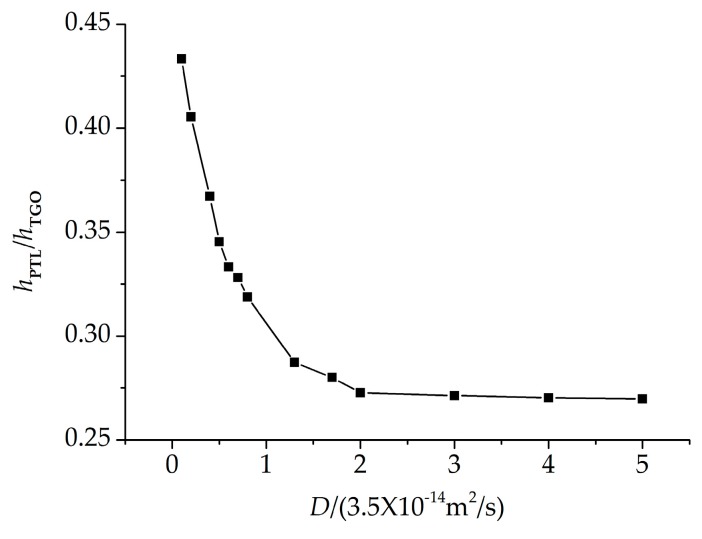
The influence of oxygen diffusion coefficient on the growth of PTL.

It is obvious that oxygen diffusion plays an important role in the growth of the PTL. To investigate the effect of oxygen diffusion on the PTL growth, different oxygen diffusion coefficients (*D* in Equation (2)) as listed in Table 3 are considered. The corresponding phase transition proportions *h*_PTL_/*h*_TGO_ can be obtained by numerical calculations, as plotted in Figure 6.

It is shown that with the oxygen diffusion coefficient increasing, the phase transition proportion decreases. The diffusion coefficient describes the rate of diffusion, which is the volume of oxygen diffusing through a unit area per unit time. To explain the oxygen diffusion effect on the PTL growth, a representative zone between TGO and BC layer is selected as shown in Figure 3. With larger oxygen diffusion coefficient *D*, more *O*^2−^ interacts with Al to form Al_2_O_3_ in unit time, which increases the oxidation rate. More Al in the representative zone will be oxidized to Al_2_O_3_, causing *ξ* increasing and the thickness of PTL *h*_PTL_ decreasing, respectively. As a result, larger *D* leads to smaller phase transition proportion *h*_PTL_/*h*_TGO_.

### 4.2. Stress State in the Phase Transition Layer

In this part, the influence of the presence of PTL on the stress state in TBCs is firstly investigated. Then, the stress states in the PTL with the constitutive equations based on the Reuss and Voigt models are studied, respectively. As the stress in *x*_2_ direction is one of the main factors leading to TBCs crack, this work is mainly aimed at exploring the stress component *σ*_22_ in TBCs.

**Figure 7:**
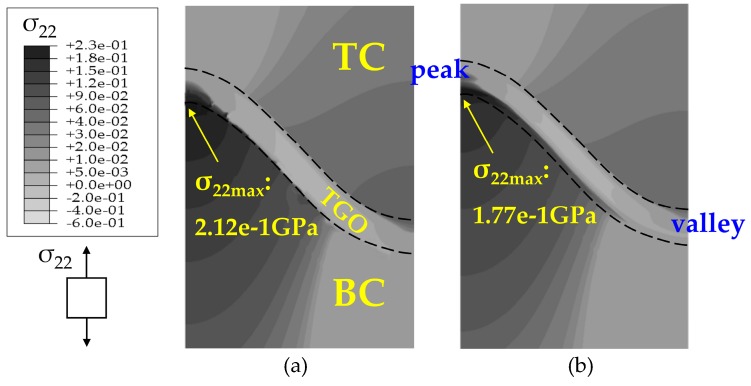
Contour plots of the stress component *σ*_22_ at 1100 °C after 200 h oxidation (*D* = 3.5 × 10^−14^ m^2^/s) based on the Reuss model: (**a**) without PTL (*ξ*_PTL_ = 1); and (**b**) with PTL (0 < *ξ*_PTL_ < 1).

**Figure 8:**
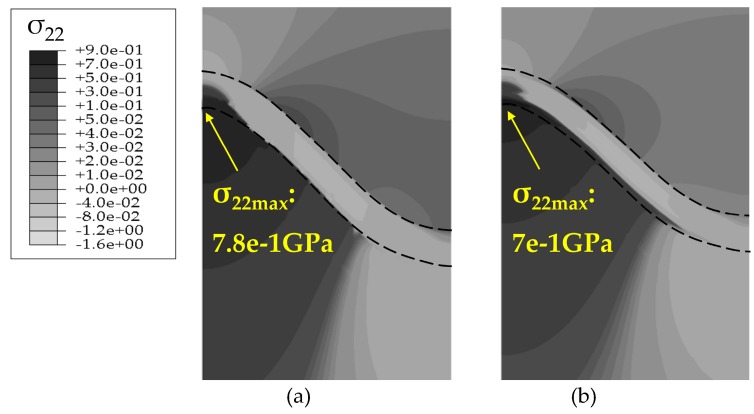
Contour plots of the stress component *σ*_22_ at 20 °C after 200 h oxidation (*D* = 3.5 × 10^−14^ m^2^/s) based on the Reuss model: (**a**) without PTL (*ξ*_PTL_ = 1); and (**b**) with PTL (0 < *ξ*_PTL_ < 1).

The stress states based on the Reuss model after 200 h oxidation are summarized in Figure 7 and Figure 8. The development of stress *σ*_22_ at the high temperature of 1100 °C is induced by the deformation associated with TGO growth. At the oxidation stage, the volume expands as the TGO forms, thus, a considerable compressive growth stress generates in the TGO layer during oxidation, leading to the out-of-plane displacement of the TGO layer. This out-of-plane displacement enhances the wrinkle of the TGO layer. On one hand, the upward bulging deformation in the peak region (as shown in Figure 7) of the TGO layer causes compression on the TC-TGO interface, as well as tension on the BC interface while, on the other hand, downward extruding deformation in the valley region of TGO layer produces the opposite effect on stress development. Thus, tensile stress accumulates at the peak of the PTL, and compressive stress at the valley of the PTL [13]. During the cooling stage, there are three factors affecting the interface stress generation, usually considered is the mismatch of thermal expansion coefficient (CTE) among TC, TGO, and BC layers Since the CTE of TGO layer is the smallest in these three layers, deformation compatibility among TC, TGO and BC layer induces compressive stress in TGO layer [20]. Then, the difference to the CTE of the substrate causes further shrink deformation of the TGO in the *x*_1_ direction [21]. Associated with the temperature dependence of the Young’s modulus [17], the compressive stress in the TGO layer are further amplified, which enhances the out-of-plane displacement of TGO. Consequently, the significant change in the magnitude of interface stress will be observed in Figure 8.

With the same TGO thickness, comparisons of stresses without PTL and with PTL are made, as shown in Figure 7 and Figure 8. Figure 7a and Figure 8a are the models without PTL (i.e., *h*_PTL_ = 0, see Figure 3), which is with the assumption that the Al_2_O_3_ dimensionless molar fraction in the PTL *ξ*_PTL_ = 1, so the PTL disappears totally and converts to be the Al_2_O_3_ layer, while the TGO with PTL in Figure 7b and Figure 8b consist of the the Al_2_O_3_ layer and the PTL (i.e., *h*_PTL_ > 0, see Figure 3). Therefore, the equivalent Young’s modulus without the PTL (*ξ*_PTL_ = 1) is larger than that with the PTL (0 < *ξ*_PTL_ < 1). As a result, the stresses without the PTL appear larger, as illustrated in Figure 7 and Figure 8.

**Figure 9:**
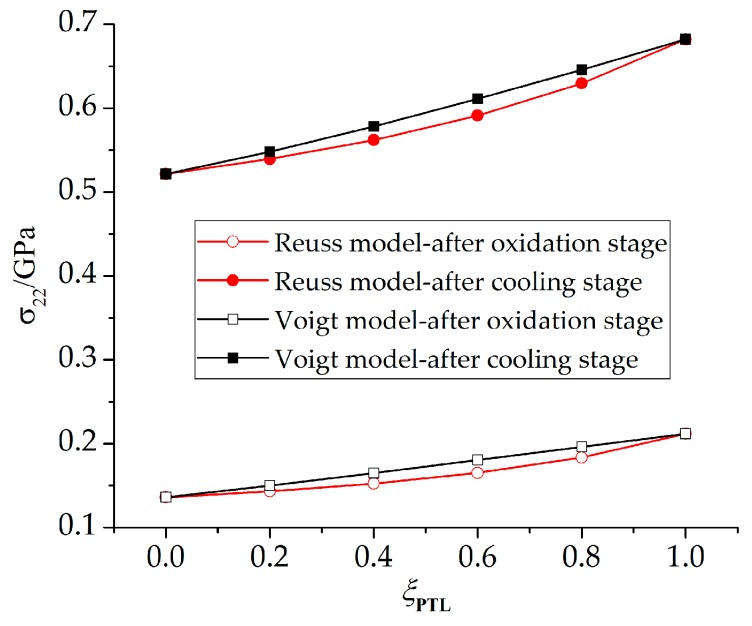
The maximum stress component *σ*_22_ at peak position based on the Reuss and Voigt models with a variation of *ξ*_PTL_ (*D* = 3.5 × 10^−14^ m^2^/s).

To study the effect of the PTL on the stress state in detail, more calculations are conducted with *ξ*_PTL_ valuing 0, 0.2, 0.4, 0.6, 0.8, 1. The maximum stresses at the peak position are extracted, as shown in Figure 9. The results show that as *ξ*_PTL_ increases, the stresses based on the Voigt model increase linearly, while the stresses based on the Reuss model increase non-linearly, and the stresses based on the Voigt model are higher than those based on the Reuss model. As in [12], in the two-phase composite materials, the equivalent Young’s modulus based on the Voigt model represents the upper bound among various homogenization models in a linear manner, while the equivalent Young’s modulus based on the Reuss model is non-linear and shows the lower bound.

From the above results, it can be found that as an Al_2_O_3_ and Al co-existent layer, the PTL affects the stress states in TBCs. In the following part, the stresses in the PTL based on the Reuss and Voigt models will be further investigated.

**Figure 10:**
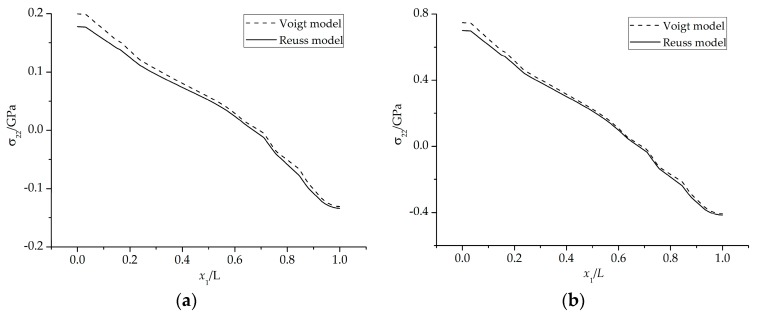
Stress *σ*_22_ in the PTL after 200 h oxidation (*D* = 3.5 × 10^−14^ m^2^/s): (**a**) at 1100 °C; and (**b**) at 20 °C.

The stresses component *σ*_22_ on the interface with *ξ*_PTL_ = 30% in the PTL are plotted in Figure 10. The horizontal axis origin is set to be the point on the finite element model where *x*_1_ = 0 and *x*_2_ = 0 (see Figure 4), and *x*_1_ represents the coordinate values of the points on the above interface in *x*_1_ direction and *L* is the length of the FE model. It is found that the stresses based on the Reuss and Voigt models have very similar distribution form but different values. The stresses based on the Voigt model is generally higher than those of Reuss model. The reason lies in that, among the homogenization models, the Voigt and Reuss models represent the upper and lower bound, which demonstrates that the equivalent Young’s modulus is the largest obtained by the Voigt model, while the smallest is by the Reuss model under the same volume fraction of inclusions. Therefore, under the same growth strain of TGO, the stresses based on the Voigt model are larger.

**Figure 11:**
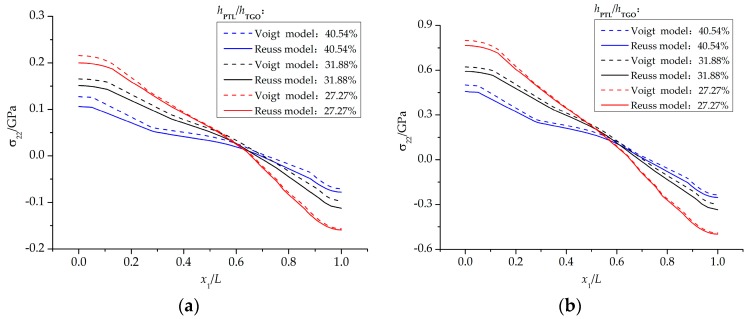
Influence of different phase transition proportions *h*_PTL_/*h*_TGO_ on the difference of stresses caused by the two constitutive equations: (**a**) at 1100 °C; and (**b**) at 20 °C.

**Figure 12:**
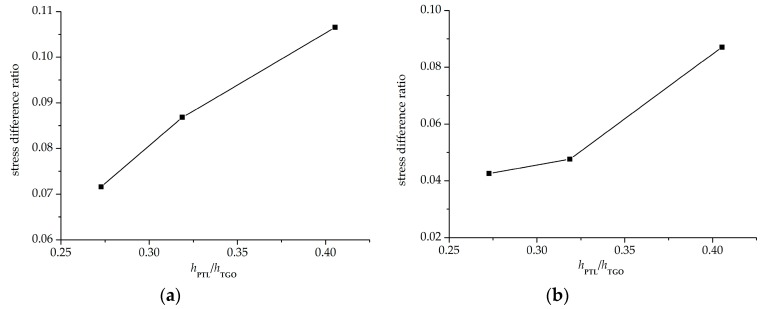
Stress difference ratio versus the phase transition proportion *h*_PTL_/*h*_TGO_: (**a**) at 1100 °C; and (**b**) at 20 °C.

However, due to the very thin PTL, the stress difference caused by the two constitutive equations mentioned above is generally very small. In the following part, more numerical calculations are conducted to explore the effect of the PTL growth on stress development. Different phase transition proportions *h*_PTL_/*h*_TGO_ coming from Figure 6 are applied in this section. The stresses on the interface of *ξ*_PTL_ = 30% under three phase transition proportions (*h*_PTL_/*h*_TGO_ = 27.27%, 31.88%, and 40.54%) are shown in Figure 11. A small *h*_PTL_/*h*_TGO_ means that the Al_2_O_3_ layer takes a large part of the TGO layer, so the equivalent Young’s modulus is larger. As a result, the stresses on the interface become greater with a decreasing *h*_PTL_/*h*_TGO_. Moreover, due to the CTE mismatch among TBCs and the effect of substrate shrink on the further deformation of TGO, the stresses on the interface after the cooling stage are higher than those during oxidation stage. Figure 12 presents the stress difference caused by the Voigt-based and the Reuss-based constitutive equations with different phase transition proportions. Here, representative points with the maximum tensile stress on the interface under three phase transition proportions are selected. It is shown that on the case of *h*_PTL_/*h*_TGO_ = 40.54%, the difference of stresses caused by the two constitutive equations seems the biggest, and gets smaller with decreasing *h*_PTL_/*h*_TGO_. Accordingly, under larger PTL thickness in the TGO layer, the effect of different constitutive relationships of PTL on the interface stress difference is more evident.

**Figure 13:**
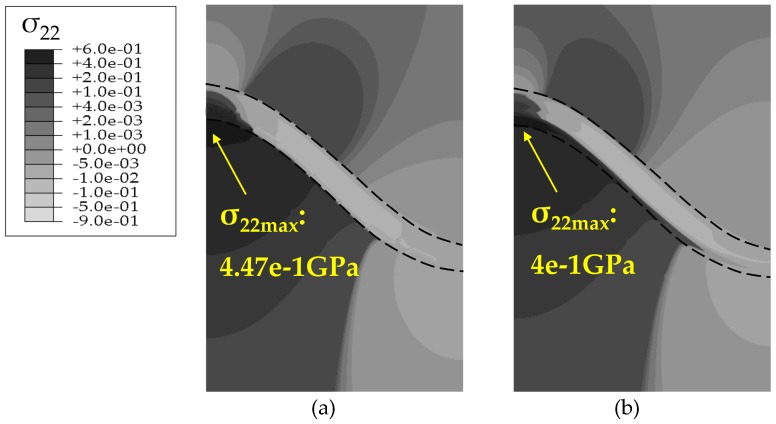
Contour plots of the stress component *σ*_22_ in the *x*_2_ axis at 20 °C with a stress-free state at the end of oxidation (*D* = 3.5 × 10^−14^ m^2^/s): (**a**) without PTL (*ξ*_PTL_ = 1); and (**b**) with PTL (0 < *ξ*_PTL_ < 1).

**Figure 14:**
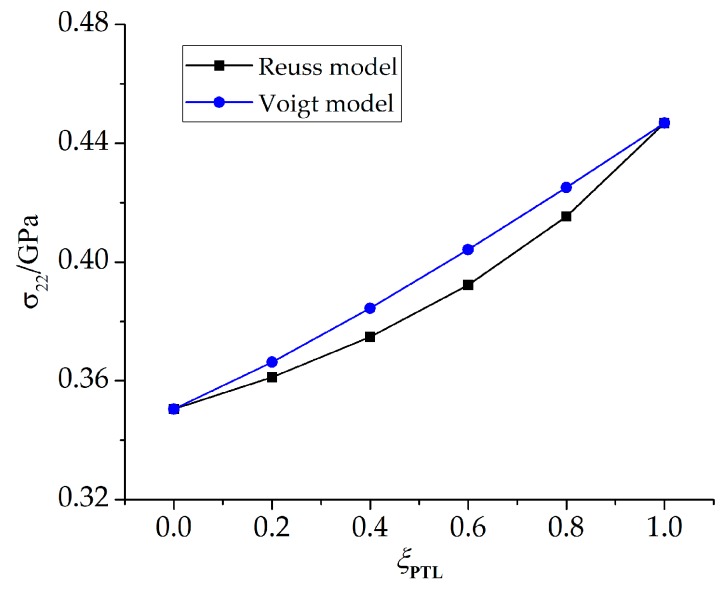
The maximum stress component *σ*_22_ at peak position based on the Reuss and Voigt models with variation of *ξ*_PTL_ (*D* = 3.5 × 10^−14^ m^2^/s) after the cooling stage (at 20 °C) with a stress-free state at the end of oxidation.

**Figure 15:**
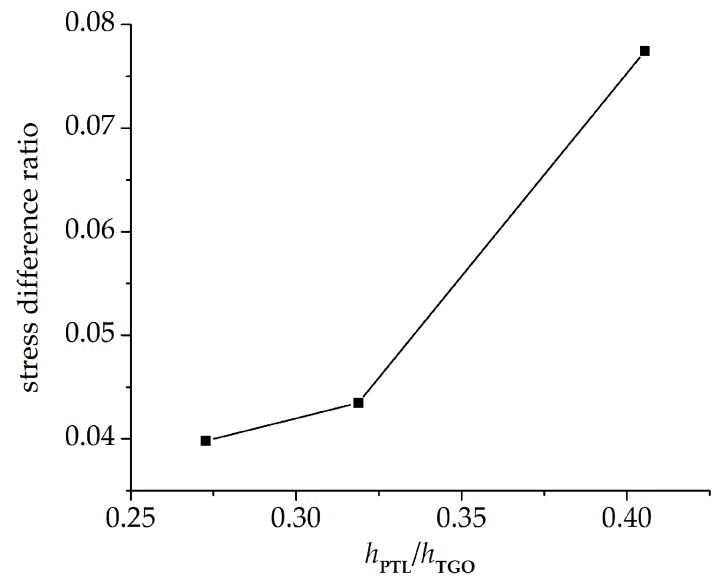
Stress difference ratio versus the phase transition proportion *h*_PTL_/*h*_TGO_ after the cooling stage at 20 °C with a stress-free state at the end of oxidation.

To disentangle the effects of TGO growth duringthe oxidation stage, more calculations are made assuming a stress-free state at the end of oxidation stage, and stresses on the cooling stage are paid attention. The influence of the PTL on the stress state is investigated. A comparison of stresses with PTL and without PTL is made as shown in Figure 13. As discussed above, the stress without PTL (*ξ*_PTL_ = 1) is higher due to the larger equivalent Young’s modulus. As well, the cooling stresses based on the Reuss and Voigt models with *ξ*_PTL_ variation are obtained, as shown in Figure 14. The stresses based on the Voigt model show increasing linearly as *ξ*_PTL_ increases, while showing a non-linear increase based on the Reuss model, which is consistent with the results that considering the effect of TGO growth during oxidation stage, as shown in Figure 8. Furthermore, the stresses without the effect of TGO growth in Figure 14 are lower than with the effect of TGO growth. Finally, it shows that with the thickness ratio of the phase transition layer in the TGO increasing, the stress difference caused by different constitutive models gets larger, as illustrated in Figure 15.

## 5. Conclusions

This work studies the stress development in the phase transition layer. Based on a diffusion-oxidation reaction model and the Reuss model-based constitutive equation, a user-defined subroutine in finite element ABAQUS code is developed. Numerical calculations have been conducted to study the effect of oxygen diffusion on the growth of the phase transition layer. Meanwhile, the stresses in the PTL with the constitutive equations based on two limiting homogenization methods— the Voigt model and the Reuss model—are also investigated, respectively. A further numerical analysis is then carried out to explore the influence of the phase transition growth states on the difference of stresses caused by different constitutive equations, which may provide a reference for further research on the stress development in TBCs. The results show that:

- A large oxygen diffusion coefficient increases the oxidation rate of metal material in the phase transition layer, and leads to a smaller part of the PTL taking part in the TGO thickness.
- The stresses in the PTL based on the Reuss model are slightly smaller than those of the Voigt model. With the Al_2_O_3_ dimensionless molar fraction in the PTL *ξ*_PTL_ increasing, the stresses based on the Voigt model increase linearly, while the stresses based on the Reuss model increase non-linearly. Meanwhile, with the thickness ratio of phase transition layer in the TGO increasing, the stress difference caused by different constitutive models becomes more evident.

This study investigates the influence of different constitutive models on stress prediction in TBCs, with the assumption that the TC, TGO, and BC layers are all considered as linear elastic materials. Further research may consider a material’s non-linear (i.e., plastic and creep) behavior in studying the influence of constitutive models on stress prediction.

## Figures and Tables

**Table 1:** The temperature-dependent mechanics parameters.

T/°C		20	200	400	600	800	1000	1100
**TC**	*E*/GPa [16]	48	47	44	40	34	26	22
	*ν* [16]	0.1	0.1	0.1	0.11	0.11	0.12	0.12
	*α* (10^−6^/°C) [16]	9.7	9.8	9.9	9.9	10	10.1	10.1
**BC**	*E*/GPa [16]	200	190	175	160	145	120	110
	*ν* [16]	0.3	0.3	0.31	0.31	0.32	0.33	0.33
	*α* (10^−6^/°C) [16]	12.3	13.2	14.2	15.2	16.3	17.2	17.7
**TGO**	*E*/GPa [16]	400	390	380	370	355	325	320
	*ν* [16]	0.23	0.23	0.24	0.24	0.25	0.25	0.25
	*α* (10^−6^/°C) [16]	8	8.2	8.4	8.7	9	9.3	9.5
**Substrate**	*α* (10^−6^/°C) [16]	14.8	15.2	15.6	16.2	16.9	17.2	17.6

**Table 2:** The diffusion and oxidation parameters.

	Parameter	Value
oxygen diffusion in TC	*D*_TC_/(m^2^/s)	∞
oxygen diffusion in BC/TGO [17]	*D*_BC_ = *D*_TGO_/(m^2^/s)	3.5 × 10^−14^
Reference value for the diffusion-oxidation model	*A* [18]/(mol/m^3^)	0.24 × 10^6^
	*γ* [19]/(m^3^/(mol·s))	1.25 × 10^−4^

**Table 3:** Values of oxygen diffusion coefficient adopted in the analysis.

Parameter	Value												
*D* (3.5 × 10^−14^ m^2^/s)	0.1	0.2	0.4	0.5	0.6	0.7	0.8	1.3	1.7	2	3	4	5
